# Synthesis, Crystal Structure, and Conductivity of a Weakly Coordinating Anion/Cation Salt for Electrolyte Application in Next-Generation Batteries

**DOI:** 10.1021/acs.accounts.2c00584

**Published:** 2023-02-22

**Authors:** Ghislain Mandouma, Journee Collins, Darrian Williams

**Affiliations:** Department of Natural Sciences, Albany State University, 504 College Drive, Albany, Georgia 31763, United States

## Abstract

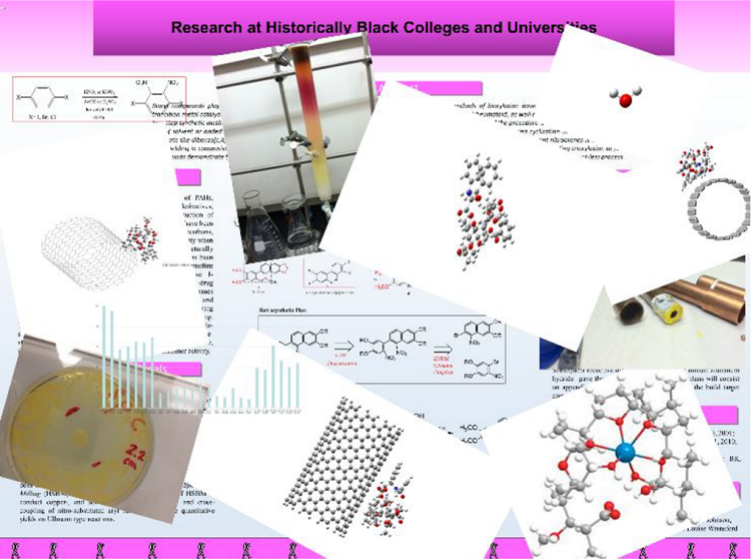

Research at historically black colleges and universities (HBCUs) started with humble beginnings by G. W. Carver at Tuskegee Institute AL, the nation’s first HBCU. He is now remembered as the man who transformed one crop, peanuts to more than 300 useful products such as food, beverages, medicines, cosmetics, and chemicals. However, research was not the focus of most of the newly founded HBCUs to provide, primarily, liberal arts education and training in agriculture for the black minority. HBCUs remained segregated, lacking facilities such as libraries and scientific/research equipment comparable to those at traditionally white institutions. While the Civil Rights Act of 1964 heralded the dawn of “equal opportunity” and progressive desegregation in the South, many public HBCUs had to close or merge with white institutions due to loss of funding and/or students. In order to remain competitive in enrollment and financial support of the best talents, HBCUs have been expanding their research and federal contracts by working in collaboration with research-intensive institutions and/or minority-serving institutions (MSIs). Albany State University (ASU), an HBCU with a great tradition of in-house and extramural undergraduate research, has partnered with the laboratory of Dr. John Miller at Brookhaven National Laboratory (BNL) to offer the best training and mentorship to our undergraduates. Students synthesized and performed conductivity measurements on a new generation of ion-pair salts. One of these constitutes, potentially, a nonaqueous electrolyte for the next generation of high-energy-density batteries owing to its electrochemical properties.

The quest for rechargeable batteries with greater energy density and capable of shorter recharge time at the “pump” for electrical vehicles (EVs) is leading the development of electrolytes with higher ionic mobility and greater limiting conductivity. In order to achieve high energy density, it is vital for an electrolyte to be electrochemically stable while operating at high voltages.

The development of a weakly coordinating anion/cation electrolyte for energy storage applications offers a challenge of technological significance. This class of electrolytes is advantageous for the investigation of electrode processes in low-polarity solvents. The improvement arises from the optimization of both ionic conductivity and solubility of the ion pair formed between a substituted tetra-arylphosphonium (TAP^R^) cation and tetrakis-fluoroarylborate (TFAB), a weakly coordinating anion. The chemical “push–pull” between cation and anion affords a highly conducting ion pair in low-polarity solvents such as tetrahydrofuran (THF) and *tert*-butyl methyl ether (TBME). The limiting conductivity value of the salt, namely, tetra-*p*-methoxy-phenylphosphonium-tetrakis(pentafluorophenyl)borate or TAP^R^/TFAB (R = *p*-OCH_3_), is in the range of lithium hexafluorophosphate (LiPF_6_) used in lithium-ion batteries (LIBs). This TAP^R^/TFAB salt can improve the efficiency and stability of batteries over those of existing and commonly used electrolytes by optimizing the conductivity tailored to the redox-active molecules. LiPF_6_ dissolved in carbonate solvents is unstable with high-voltage electrodes that are required to achieve greater energy density. In contrast, the TAP^OMe^/TFAB salt is stable and has a good solubility profile in low-polarity solvents given its relatively great size. And it constitutes a low-cost supporting electrolyte capable of bringing nonaqueous energy storage devices to compete with existing technologies.

## Key References

- BoucardR.; ReaganP.; MandoumaG.Synthesis and Conductivity Studies of Tetraarylphosphonium Salts as Potential Electrolytes in Advanced Batteries. Int. J. Innov. Ed. Res. (IJIER)2018, 6, 116–12310.31686/ijier.vol6.iss2.955.^1^*This preliminary electrochemical investigation of a series of tetraarylphosphonium-based salts in low-polarity solvents established their high conductivity in those media*.
- WhitfieldZ.; BibbsJ.; MandoumaG.; MillerJ.; BirdM.; ManiT.; WilsonR.Development of Tetraarylphosphonium/Tetrakis(pentafluorophenyl)borate (TAP^R^/TFAB) salts as non-aqueous electrolytes for organic redox flow batteries. Int. J. Innov. Ed. Res. (IJIER).2019, 7, 492–49810.31686/ijier.vol7.iss12.2098.^2^*Ion pairs constituted of weakly coordinating cations and anions were synthesized and characterized as potential electrolytes for redox flow batteries (RFBs) due to their excellent conductivity in low-polarity organic solvents*.

## 1 Introduction

HBCUs were founded, first in the United States South following the end of the Civil War and the abolition of slavery, to help former slaves adjust to freedom. Most HBCUs provided a basic liberal arts education and trained students for careers as teachers, ministers, or missionaries. Others focused on preparing students for industrial or agricultural occupations such as Booker T. Washington’s Tuskegee Institute, founded in 1881. They thrived in the racially segregated South where it was nearly impossible for black students to enroll anywhere other than at HBCUs. With the desegregation of schools and institutions of higher learning in the mid-1960s, funding of HBCUs dipped as African Americans were no longer restricted to study at HBCUs.^3^ Today, 1 out of every 10 black college students attends an HBCU, and increasing numbers of whites and Latinos also attend these institutions, according to data from the National Center for Education Statistics.^4^ Currently, there are 105 HBCUs nationwide that are an important part of the U.S. education system. They provide a sizable pool of talents that are crucial to the nation’s diminishing science, technology, engineering, and mathematics (STEM) workforce, according to Forbes magazine.^5^ The National Academies of Sciences, Engineering, and Medicine has, in a new report, stressed the need for policymakers and education leaders to strengthen STEM programs and attainment of degrees at the nation’s minority-serving institutions (MSIs). HBCUs constitute some of the most important MSIs as they are still the largest contributors of minority graduates to enter the workforce in the United States.^6−8^ One in six African Americans earning a bachelor’s degree graduates from an HBCU. At Albany State University (ASU), a rigorous training of STEM majors is conducted through a combination of classical laboratory courses and sponsored research projects. We also collaborate with research-intensive institutions nationwide to supplement what we and other HBCUs lack: major research instrumentation and highly trained research postdoctoral fellows.

It is now recognized that systemic racism has created barriers for people of color, including hostile working conditions and a lack of adequate funding.^9−11^ In order to carry on with their mission, it became crucial for most HBCUs to forge alliances with progressive institutions which promote shared values of diversity, equity, and inclusion in their efforts at changing policies and procedures to be more equitable for all,^12^ especially in terms of access to funds and equipment. Clearly, the nation’s HBCUs need adequate funding to overcome all kinds of financial peril and be able to compete. Universities must compete to attract students, and that is done by producing new knowledge through research. Nowadays, universities use research as the cornerstone of their strategic plan for growth by taking in more money in federal research and contracts than from tuition and fees charged to students.^13^

As an HBCU science faculty member, my efforts at securing research funding led to important, career-defining collaborations with several faculty members and departments from majority institutions. One early effort, now a long-standing collaboration between ASU and Furman University (FU), started in 2010 when my team of ASU students and faculty was invited to submit our applications to the 10-week NSF-funded summer REU (Research Experience for Undergraduates) program at FU under the leadership of Professor Tim Hanks (now Chair, FU) and Dr. Karen Buchmueller. This REU program included teams of minority faculty and students from several HBCUs widely distributed in the U.S. The summer REU provided learning opportunities for both students and faculty involved in the program. Weekly seminars allowed students to present their work in progress as well as actively participate in the Q&A sessions. Students were encouraged to explore the best possible routes of accomplishing their research projects by the faculty and/or their own peers. The final poster session brought the best from students in term of their creativity, learning, and readiness to answer questions from an audience of experts in the field. The exchanges remained cordial and aimed at improving one another. The REU offered more than laboratory experiences and research-grade instrumentation for ASU students. A weekly career development series of talks by prominent scientists and professionals pointing to different career paths served to convince one of my students to pursue a Ph.D. This, in fact, was one of the understated goals of the REU: to provide an opportunity for underrepresented students to create their own social network of peers in the same discipline but from different backgrounds while experiencing research on a daily basis. Over the 10-week period, working relationships between students and faculty were established through hands-on training and mentoring which could last a lifetime. My students and I were associated with REU-related activities and conferences. My own research program at ASU grew tremendously from data produced at the REU. These turned into publications in peer-reviewed journal articles and publications with my students.^14,15^ The ASU–FU relationship has become a community of individuals that seeks to support academic success by meeting students’ needs individually, including in their search for employment postgraduation.

The Department of Energy Visiting Faculty Program (DOE-VFP) was my next source of funding. The VFP is but one aspect of the portfolio of programs at DOE’s Brookhaven National Laboratory (BNL) where the VFP is conducted every summer. Hundreds of students and faculty are brought to the campus in Upton, NY every summer. This becomes obvious on the last day of the summer internship when awards are handed out. I became aware only afterward that a recruiting process has been planned and carried out months before. I was introduced to Dr. Noel Blackburn, the Director of BNL’s Office of Education Program (OEP), while visiting Argonne National Laboratory in summer 2015. An opportunity to visit BNL, NY in the fall of 2015 was extended to a group of faculty, during which we visited several faculty/laboratory directors in the chemistry department. From those interactions were born collaborations between BNL and HBCUs. I was invited to submit an application to the VFP program in collaboration with the group of Dr. John Miller, and subsequently awards were granted to me and two accompanying students. Our summer appointment at BNL lasted three summer sessions and involved six hard-working students who conducted salt syntheses (under my supervision at ASU and BNL) and electrochemical characterization (with BNL postdoctoral researchers). It is from the 3-year collaboration between ASU and BNL that we were able to gather valuable data on the novel TAP^R^/TFAB supporting electrolytes for next-generation batteries and other forms of electrochemical energy storage. With the research support and mentorship of Dr. John Miller and his laboratory staff, my research group at ASU received funding from the National Science Foundation (NSF) in the form of a Research Initiation Award (RIA). This grant provided us with the opportunity to train even more students in undergraduate research at ASU while building more research capacity in mostly rural Southwest GA. It was most gratifying that my students moved on to graduate and medical schools just as I had hoped they would at the end of their ASU–BNL experience. These are examples of what faculty at HBCUs must sometimes do in order to fund their research program while helping to provide students with first-class research experience. In this Account, we describe those efforts leading to the discovery of a new generation of supporting electrolytes endowed with high ionic conductivity. This makes them suitable as electrolytes for next-generation high-energy-density batteries and other electrochemical energy storage (EES) systems.

The current energy crisis and climate change concerns have, once more, highlighted the need for industrialized nations to transition away from coal and fossil fuels to “green” and renewable sources for energy production. Solar energy and wind power are promising renewable sources despite their intermittent nature while geothermal energy and hydroelectricity, plagued with issues of limited availability and/or negative ecological impact, have limited future prospects.^16−18^ As society becomes increasingly dependent on these renewable sources of energy, a combination of the said sources and adequate storage systems can bring about energy independence by mitigating the intermittent nature of these resources.^19,20^ “Smart grids” combine intermittent renewable energy production and energy storage such as Li-ion battery plants (powerwalls) and redox-flow batteries. These can be adapted to the generator unit and suited for large-scale storage of wind and solar electricity. Electrochemical energy storage (EES) systems are necessary for achieving the full potential of integrating solar and wind power as part of the electric power grid. The development of novel systems capable of storing and releasing when needed excess energy for immediate use offers promises of much improved energy density needed to power everything from high-mileage electric vehicles (EVs) to energy-hungry homes and businesses while also electrifying and bringing development to large parts of the world at low cost. Battery technology is one of the important components in renewable energy to store produced energy.^21−23^

Batteries are made of three parts: an anode which has an excess of electrons, a cathode lacking electrons, and an electrolyte. While electrons are traded between the anode and the cathode in the external circuit, positive ions are transported in the electrolyte from the anode to the cathode through a separator that controls the ionic flow. In rechargeable batteries, the electron flow between the electrodes can be reversed thousands of times with a charger. The chemical stability of electrodes must be preserved in the chosen electrolyte and at the operating voltage and temperature by keeping intact the solid electrolyte interphase (SEI) where the electrolyte interacts with the electrodes. In order to achieve a higher current (energy) density, all three components must be compatible to perform optimally within the same “window”. Ideally, an electrolyte should be a good ionic conductor to facilitate ion transport, and it should possess a wide electrochemical window. This prevents degradation of the electrolyte within the voltage range of the working electrodes. A good electrolyte should also be unreactive to other cell components and thermally stable with both the melting and boiling points well outside the operating temperatures. It must have low toxicity and be safe for the environment. It must be made, preferably, from sustainable chemicals using earth-abundant elements and synthetic processes of as small an impact as possible and at low total cost for materials and production. Excellent reviews have appeared on the state of different batteries and their merits and on how to improve them in terms of energy density output.^24−28^

Nowadays, the lithium-ion battery (LIB) has become ubiquitous among EES, being essentially a “bank” of energy that can be tapped to release it whenever needed. The LIB and Li-ion technology became possible with the first successful intercalation of lithium ions in graphite sheets during the 1990s.^29^ Although the LIB is now a prominent technology, its utilization of metals poses the problem of long-term stability, disposal, and handling. The electrolyte frequently used in the LIB, LiPF_6_, is toxic, and lithium is highly reactive with moisture. Also notable is the production of dendrites that can impact the thermal stability of the LIB over a long period. There is also the incompatibility of the electrolyte solution of LiPF_6_ in carbonate solvents with high-voltage cathodes that are required for achieving higher energy density. LiPF_6_ electrolyte dissolved in carbonates is known to decompose above 4.9 V.^30^

In order to address these pressing issues of the LIB safety and stability, other alternative electrolytes need to be explored. The introduction of fluorinated carbonate solvents provided stability and better performance for LiPF_6_ in high-voltage LIBs. However, the decomposition of LiPF_6_ by trace moisture remains an issue which causes the degradation of cathode material and a loss of battery capacity during long-term cycling.^31−34^ Presumably, fluorine substitution of hydrogen in carbonate solvents lowers the levels of both the HOMO and LUMO, contributing to higher reduction and oxidation potentials of the solvent.

Hill and Mann reported the first reversible one-electron process in their oxidation of ruthenocene using tetrabutylammonium tetrakis[3,5- bis(trifluoromethyl)phenyl]borate, a weakly coordinating anion (WCA), as a noncoordinating electrolyte. They underlined two important factors due to the WCA in the stability of electron-deficient metal complexes, namely, their low nucleophilicity and the increased solubility of their salts in low-polarity solvents. WCA-based salts became attractive as supporting electrolytes for oxidative electrochemical processes.^35^ Mullen and Floudas developed a class of WCAs and weakly coordinating cations (WCCs) using tetraarylborate anions and tetraphenylphosphonium cations, respectively.^36^ The association constants *K*_*A*_ of these ion pairs were found to be lower than those of conventional electrolytes, thus making weakly coordinating anion/cation ion pairs more conductive than conventional electrolytes.

**Figure 1:**
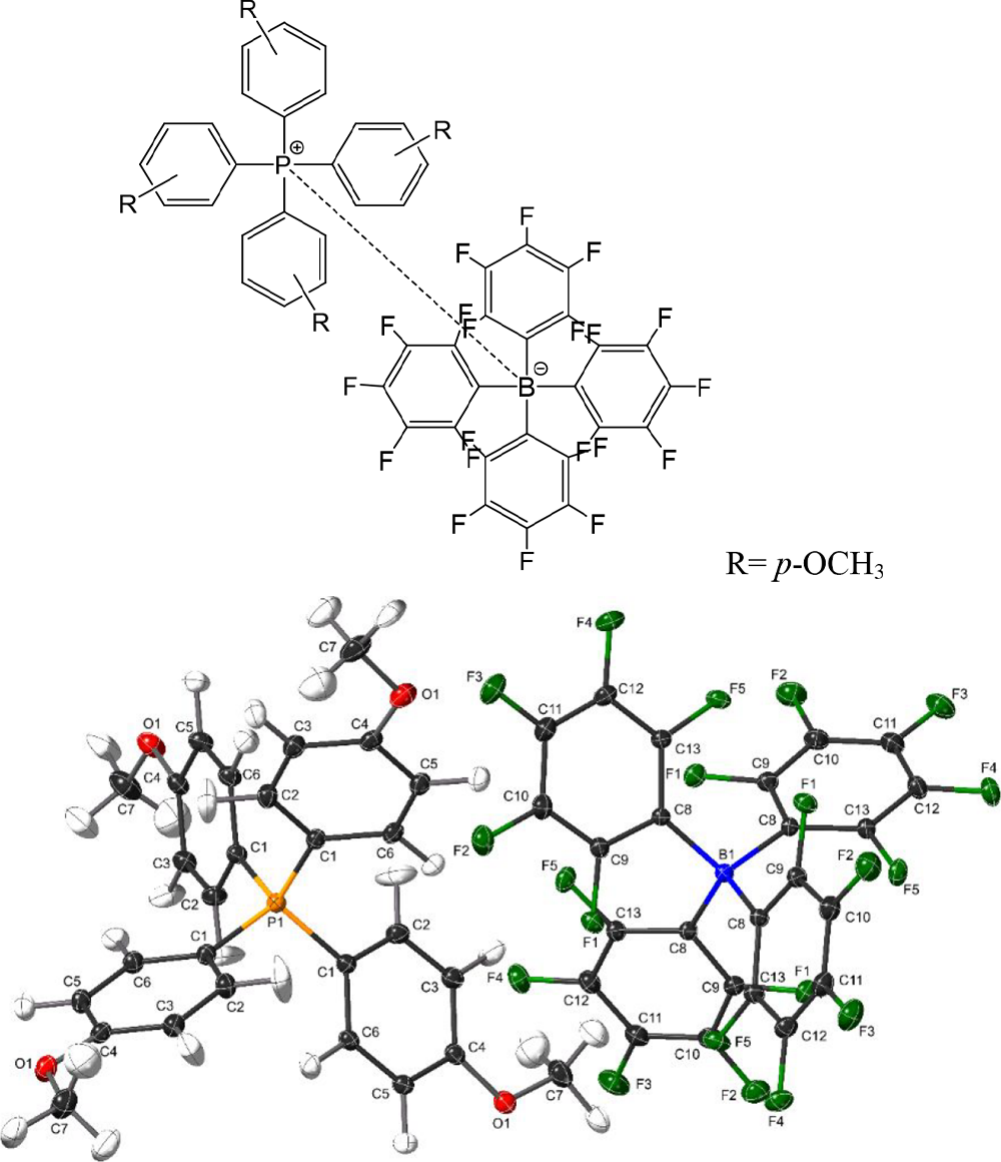
Structure of tetraarylphosphonium/tetrakis(pentafluorophenyl)borate or TAP^R^/TFAB (R = *p*-OCH_3_) salt and X-ray crystal structure of the ion pair showing spheres to represent H (gray), C (black), O (red), P (orange), F (green), and B (blue) atoms, respectively.

In this Account, we describe the synthesis, crystal structure, and high conductivity of a novel WCA/C or TAP^R^/TFAB salt (ZW1), the first of a new and versatile generation of supporting electrolytes which expands anodic applications by providing a dramatically different medium in which to generate positively charged electrolysis products. Weakly coordinating anion/cation ion-pair TAP^R^/TFAB salts have the potential to improve the efficiency and stability of the LIB and other batteries over those of existing and commonly used electrolytes by optimizing their conductivity that can be tailored to the redox-active molecules.^37^ Conductivity is a measure of the mobility of ionic species in a solvent. Low-polarity organic solvents are of great interest because of the following physical and electrochemical properties: low dielectric constant to dissociate the electrolyte salt, high fluidity for good ionic conductivity, low flammability for good safety profiles, and lower toxicity. The contribution of TAP^R^/TFAB to the thermal stability of the electrolyte solution can be significant as LiPF_6_–carbonates is known to decompose above 60 °C. The highly fluorinated TAP^R^/TFAB electrolyte is thus anticipated to be suitable for operation with high-voltage cathodes in LIB as well as other batteries. Indeed, electron-withdrawing-group-containing anions, such as TFAB, are weak nucleophiles, more weakly coordinating to their counterpart cations than conventional anions used in the LIB (e.g., PF_6_^–^) that are strongly coordinating. Computational work has shown that a strong electron-withdrawing effect of peripheral fluorine atoms in the anion contributes to significantly lower the highest occupied molecular orbital (HOMO) energy level, making its oxidation unlikely.^38,39^ Therefore, we propose a novel fluorinated WCC-WCA salt, namely, tetra-*p*-methoxyphenylphosphonium-tetrakis(pentafluorophenyl)borate or TAP^OMe^/TFAB. Its physical and electrochemical properties, including a single-crystal X-ray crystallography structure (Figure 1), are reported herein.

## 2 Results and Discussion

### 2.1 Synthesis and Structural Analysis

**Scheme 1:**
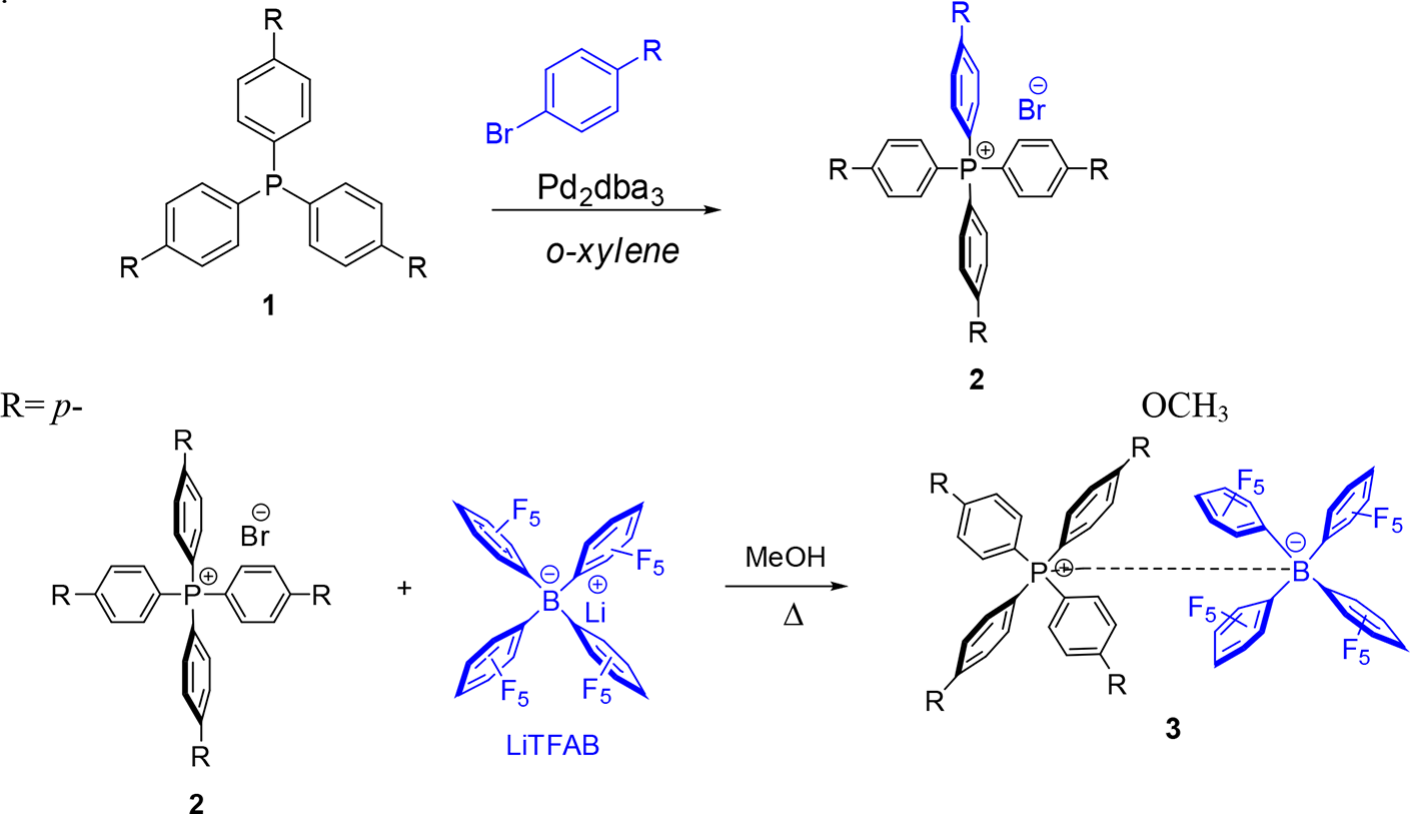
Synthesis of the Novel TAP^R^/TFAB (R = *p*-OCH_3_) Salt **3** in Two Simple Steps

The synthesis of the novel TAP^R^/TFAB (R = *p*-OCH_3_) salt was carried out in two steps. From a retrosynthetic standpoint, we envisioned that a metathesis reaction between a molar equivalent of both TAP ylide **2** and commercially available LiTFAB would yield TAP^R^/TFAB (R = *p*-OCH_3_) salt **3**, following the synthesis of phosphonium ylide **2** through a Pd-catalyzed cross-coupling reaction between an aryl halide and substituted triarylphosphines **1**.^40^ Both steps of the synthetic scheme were found to proceed rapidly, yielding products by simple filtration without lengthy purification procedures as depicted in the general Scheme 1. Substituted aryl halide (R = *p*-OCH_3_) underwent a Pd-catalyzed cross-coupling reaction with substituted triarylphosphine **1** (R = *p*-OCH_3_) in boiling xylene to afford substituted tetraarylphosphonium ylide **2**. The phosphonium ylide **2** precipitated in nonpolar xylene and was isolated by simple filtration. The subsequent metathesis reaction between the ylide **2** and lithium tetrakis(pentafluorophenyl)borate (Li^+^ TFAB^–^) resulted in the formation of lithium halide and the desired ion pair TAP^R^/TFAB (R = *p*-OCH_3_) **3**.

The formation of the salt **3** was confirmed by ^1^H, ^13^C, and ^19^F NMR analysis (Supporting Information) and by the X-ray crystal structure of the compound, which has never been reported previously. The crystal structure of **3** provides the ultimate proof of formation as well as insights into the molecular structure of the ion pair **3**. Crystallographic data for the structure reported in this Account have been deposited with the Cambridge Crystallographic Data Centre (GM-B157 in CCDC). These data can be obtained free of charge from The Cambridge Crystallographic Data Centre (www.ccdc.cam.ac.uk/structures). The purity of salt **3** was also confirmed by high-resolution mass spectrometry (Supporting Information) which unequivocally showed evidence of both the TFAB anion and TAP^OMe^ cation.

Electrochemical experiments (Supporting Information) were conducted to evaluate salt **3** as a potential electrolyte in low-polarity solvents such as tetrahydrofuran (THF) and *tert*-butyl methyl ether (TBME). The association constant *K*_*A*_ of salt **3** and limited conductivity in those media were measured with the goal of demonstrating that salt **3** constitutes a potential electrolyte in low-polarity solvents. Conductance values were obtained using an YSI model 3200 conductance meter and a conductivity cell with a cell constant of 0.1 cm^–1^. We performed those experiments to probe the conductance of a solution of synthesized electrolyte TAP^R^-TFAB (R = *p-*OCH_3_) in low-polarity solvents such as THF and TBME. Electrochemistry in these solvents is challenging due to the lack of current flow therein. However, nonpolar and low-polarity solvents can be made sufficiently conducting when weakly coordinating anions/cations, which can carry electric charges, are dissolved in these solvents. A difference in potential can then be measured in solution. Those electric charges are ions (cations and anions) formed by dissociation of a molecule. The attractive energy *E* of the cation–anion interaction is related to the dissociative energy of the thermal motion *kT* (where *k* is the Boltzmann constant and *T* is the absolute temperature) according to Bjerrum theory.^41^ This theory predicts that ions might exist separately only if their size σ_ιον_ exceeds a certain value, the so-called Bjerrum radius σ_B_. Otherwise, they would build cation–anion pairs as the thermal motion would not be able to prevent their aggregation at short distances. Morrison calculated that the diameter of ions must be larger than 28 nm in order for them to exist as free ions in low-conducting media.^42^

Our goal was to determine the association and dissociation constants of TAP^R^-TFAB (R = *p-*OCH_3_) or the TAP^OMe^ -TFAB ion pair in solutions of low-polarity solvents. The strongly electron-donating *p*-OCH_3_ group was found to enhance the limiting conductivity by increased dissociation (*K*_*D*_) of the ion pair accordingly, which is in sharp contrast to the conventional electrolyte TBA-PF_6_, which displays a greater association constant (*K*_*A*_) of 100-fold in comparison to that of TAP^OMe^-TFAB. Our data in Table 1 show a lowering of the association constant *K*_*A*_ for TAP^OMe^/TFAB in THF compared to the association constants of TBA/PF_6_ and especially TBA/TFAB. Further electrochemical experiments (provided in the Supporting Information) established the correlation between a decrease in ion pairing (lowering of *K*_*A*_) and an increase in the limiting conductivity of the ion pair in low-polarity solvents (THF and TBME).

**Table 1:** Dissociation Constant and Limiting Conductivity Values of Selected Electrolytes

aryl substituent R	dissociation constant (*K_D_*)	limiting conductivity (Λ_o_)	association constant (*K*_*A*_)
*p-*OCH_3_	2.29 × 10^–4^ M	78.6 S/mol	3.3 × 10^3^ M
LIB Electrolytes			
TBA-TFAB	1.63 × 10^–4^ M	86.2 S/mol	8.33 × 10^3^ M
TBA-PF_6_	2.86 × 10^–6^ M	86.2 S/mol	373.6 × 10^3^ M

The limiting conductivity in THF for TAP^OMe^-TFAB was measured at 78.6 S/m, which is in the range of that for tetrabutylammonium-phosphorus hexafluoride (TBA-PF_6_), the leading industrial supporting electrolyte at 86.2 S/m. Further fine-tuning in the substitution pattern of the aromatic rings of the TAP^R^ can optimize the upper limit of conductivity of TAP^R^-TFAB ion-pair electrolytes through enhanced dissociation and increased solubility of these electrolytes in low-polarity solvents. Seemingly, the methoxy electron-donating group in the *para* position has a stabilizing effect on the phosphorus cation which triggers a dissociation from the borate anion in TAP^OMe^-TFAB. In any case, the aromatic ring system of TAP^R^-TFAB ion pairs is amenable to further fine-tuning that can boost the conductance of this novel class of electrolytes in low-polarity solvents.

The reduction in association constants *K*_*A*_ from TBA^(+)^ to TAP^OMe (+)^ (with both using TFAB ^(−)^ as a counterpart anion) in THF is promising as it suggests success in designing TAP^OMe^/TFAB to be a WCC-WCA ion pair less capable of strong ion pairing. A decrease in the *K*_*A*_ value is also seen in the TBME conductivity tests, further supporting the idea that TAP^OMe^/TFAB is more weakly coordinating than TBA/TFAB. Indeed, nonpolar and low-polarity solvents can be made sufficiently conducting when weakly coordinating anions/cations are dissolved in a low-polarity solvent.^43^ Ions associate in solution to form a stable entity in media of low permittivity because Coulombic interactions are greater than the thermal energies of the “separated” ions. Increasing the size of the cation in conjunction with the already large anion can produce a “superweak” ion pair that can be dissociated in solvents of low polarity as an increase in the dielectric permittivity of the solvent raises the attractive part of the potential. Increasing the size and bulkiness of the molecular cations works to force a separation of ions. Further development of these novel electrolytes such as the TAP^OMe^/TFAB salt described herein is ongoing with additional cyclic voltammetry studies to define working electrodes. X-ray photoelectron spectroscopy (XPS) analysis and scanning electron microscopy (SEM) optical imaging are used to probe the morphology of used electrodes. Such experiments are needed to characterize the anode and the SEI formed on its surface in various electrolytes with TAP^OMe^/TFAB salt as an additive to probe for Li dendrite formation or the suppression thereof on the electrode’s SEI. Other EES systems offer tremendous opportunities as high-energy-storage systems such as redox-flow batteries (RFBs). In these, an electrolyte solution is circulated between an electrochemical cell and two tanks. The redox-active electrodes are dissolved in the electrolyte, which allows the power of the battery to be scaled up independently of its capacity. Low-polarity organic solvents show better electrochemical stability and a wider potential window than aqueous solvents, which can lead to RFBs with higher energy densities, as redox couples with an elevated voltage can be used. This represents an opportunity to test our TAP^OMe^/TFAB salt as a high-energy-density electrolyte for RFBs.

## 3 Conclusions

Electrolytes in lithium ion batteries are solutions whose function is to ferry Li^+^ ions between the electrodes. Their most important properties are stability (both thermal and hydrolytic) and high conductivity. Then, they must also display a high discharge rate and good performance at both high and low temperatures. The lithium-ion battery (LIB) was developed in the 1980s in Japan.^44^ The LIB is now ubiquitous in the EV industry and in other household small electronics as well as power tools. The success of the LIB is due, in part, to the nonaqueous electrolyte used in conjunction with high-capacity electrode materials thus enabled. However, the interactions of electrodes with electrolytes still need to be improved as dendrite formation continues to plague the LIB and the quest for even greater capacity and energy density calls for high-voltage-compatible electrolytes.^45^ The utilization of organic materials can also reduce the acquisition costs per kWh compared to cost-intensive metals used in the Li-ion batteries.

Other electrochemical energy storage (EES) systems such as redox flow batteries (RFBs) can provide flexible and scalable energy storage, whether for domestic or large-scale application as they utilize noncorrosive, safe, and low-cost organic charge-storage materials.^46,47^ There is a certain advantage with the WCA/WCCs as supporting electrolytes owing to their increased solubility in organic solvents, above inorganic salts LiPF_4_ and LiPF_6_ commonly used in Li-ion batteries. Greater solubility and higher conductivity exist with organic active materials, which represent an ideal class of materials, since their redox potentials can be fine-tuned by variation of the substituents.^48^ These TAP^R^/TFAB ion pairs are highly soluble low-polarity organic solvents, synthesized with minimal effort in high yields to enable an efficient production of large-scale systems to increase capacities and the energy-density range.

Research at an HBCU is both challenging and rewarding from an instructor’s point of view. One must accept working with fewer resources but look forward to shaping unique lives through dedicated training and mentorship. It is an adventure bringing together many partners for which the instructor is a crucial link. The instructor must be adaptable, often searching for opportunities that are extended to benefit students primarily. Sometimes it takes inquiring to discover federal and state funding and shared equipment and/or resources available to HBCUs that are located at majority institutions. It has been said that “it takes a village to train up a child”. ASU and HBCUs could not have trained our finest students who are now Ph.D.s, M.D.s, and other professionals without the additional resources provided by other institutions and federal agencies that are also devoted to students’ success.^49−51^ To do this is to fulfill the legacy of George W. Carver who said “It is simply service that measures success”.

## Supporting Information Available

Experimental details and spectroscopic data including ^1^H, ^13^C, ^19^F, and ^31^P NMR spectra and high-resolution mass spectra for all compounds (PDF)
