# Supplementary material for: Synthesis, Crystal Structure, and Conductivity of a Weakly Coordinating Anion/Cation Salt for Electrolyte Application in Next-Generation Batteries
Source: Acc Chem Res. 2023 Feb 22;56(11):1263–70. doi: 10.1021/acs.accounts.2c00584 (PMC10249345; doi:10.1021/acs.accounts.2c00584)
Supplement: Supplementary file 1 — ar2c00584_si_001.pdf [file ar2c00584_si_001.pdf]

Supporting Information

Synthesis, Crystal Structure, and Conductivity of a Weakly Coordinating Anion/Cation Salt for Electrolyte Application in Next Generation Batteries

Ghislain Mandouma[a]*, Journee Collins[a,b] and Darrian Williams[a,c]

[a] Department of Natural Sciences, Albany State University, 504 College Drive, Albany, Georgia 31763, United States.

[b] J.C.: Pharmavite LLC, 4701 Northpark Dr, Opelika, AL 36801, United States.

[c] DW: Coca Cola, 427 San Christopher Dr, Dunedin, FL 34698, United States.

*Email: gmandoum@asurams.edu

Experimental

X-ray Crystallography of ZW1 (GM-B157 in CCDC)

Experimental: The material (ZW1) was used as supplied. The data for ZW1 (GM-B157) including single crystal X-ray (**Figure S1**, **Table S1**) and crystal packing (**Figure S2**) were collected from a shock-cooled single crystal at 100(2) K on a Bruker D8 VENTURE dual wavelength Mo/Cu four-circle diffractometer with a microfocus sealed X-ray tube using a mirror optics as monochromator and a Bruker PHOTON II detector. The diffractometer was equipped with an Oxford Cryostream 800 low temperature device and used Mo radiation (λ = 0.71073 Å). All data were integrated with SAINT and a none absorption correction using SADABS was applied.[1,2] The structure was solved by dual methods using SHELXS-97 and refined by full-matrix least-squares methods against F^2 by SHELXL-2014.[3,4] All non-hydrogen atoms were refined with anisotropic displacement parameters. The hydrogen atoms were refined freely with anisotropic displacement parameters. Crystallographic data for the structures reported in this paper have been deposited with the Cambridge Crystallographic Data Centre (CCDC).[5] These data can be obtained free of charge from The Cambridge Crystallographic Data Centre via www.ccdc.cam.ac.uk/structures. This report and the CIF file were generated using FinalCif.[6]

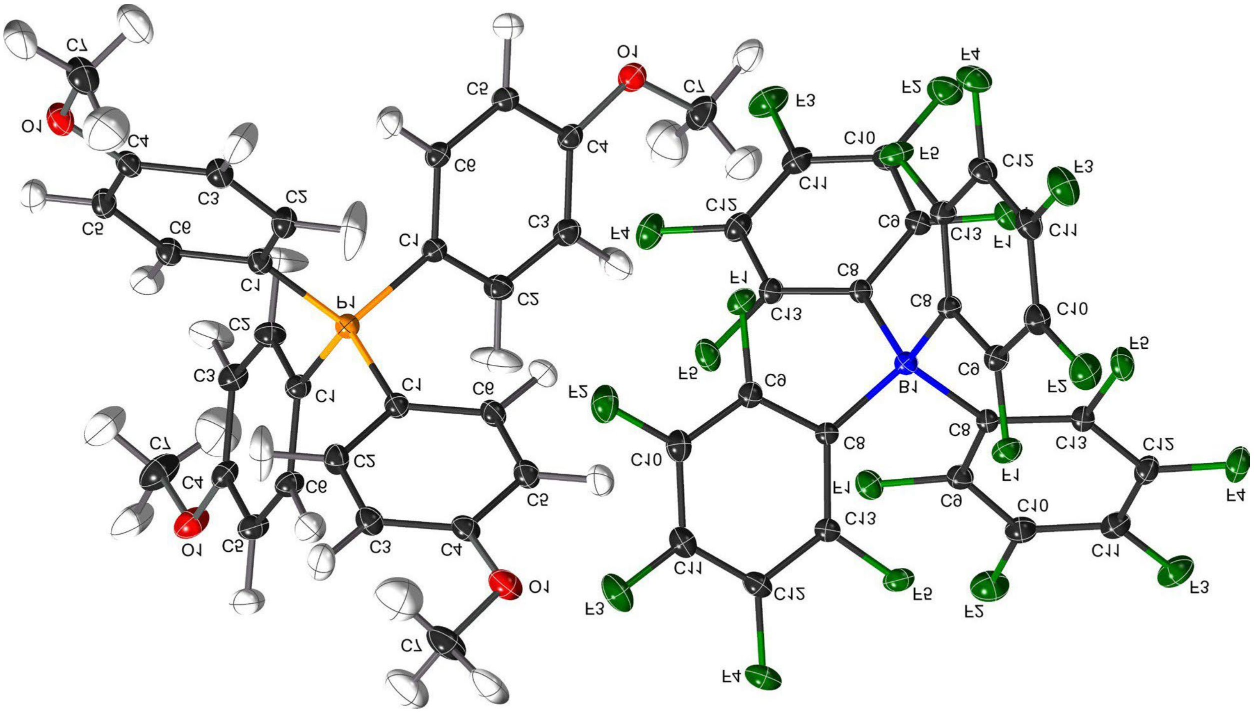

Figure S1. X-ray single crystal structure of ZW1 (GM-B157 in CCDC)

Table S1. Crystal data and structure refinement for GM-B157 (ZW1)

Empirical formula	C52H28BF20O4P
Formula weight	1138.565
Temperature [K]	100(2)
Crystal system	tetragonal
Space group (number)	IIIIIIII4 (82)
a [Å]	17.5753(3)
b [Å]	17.5753(3)
c [Å]	7.4071(2)
α [°]	90
β [°]	90
γ [°]	90
Volume [Å3]	2287.99(8)
Z	2
*ρ*calc [gcm−3]	1.653
μ [mm−1]	0.192
F(000)	1145.324
Crystal size [mm3]	0.34×0.232×0.156
Crystal colour	yellow
Crystal shape	prism
Radiation	Mo (λ=0.71073 Å)
2θ range [°]	5.96 to 71.30 (0.61 Å)
Index ranges	−16 ≤ h ≤ 28 −27 ≤ k ≤ 28 −12 ≤ l ≤ 12
Reflections collected	21645
Independent reflections	5281 *R*int = 0.0784 *R*sigma = 0.0657
Completeness to θ = 25.2417°	99.7 %
Data / Restraints / Parameters	5281/102/241
Goodness-of-fit on *F*2	1.0356
Final *R* indexes [*I*≥2σ(*I*)]	*R*1 = 0.0477 w*R*2 = 0.0949
Final *R* indexes [all data]	*R*1 = 0.0624 w*R*2 = 0.1045
Largest peak/hole [eÅ−3]	0.44/-0.32
Flack X parameter	0.05(8)
Extinction coefficient	0.0033

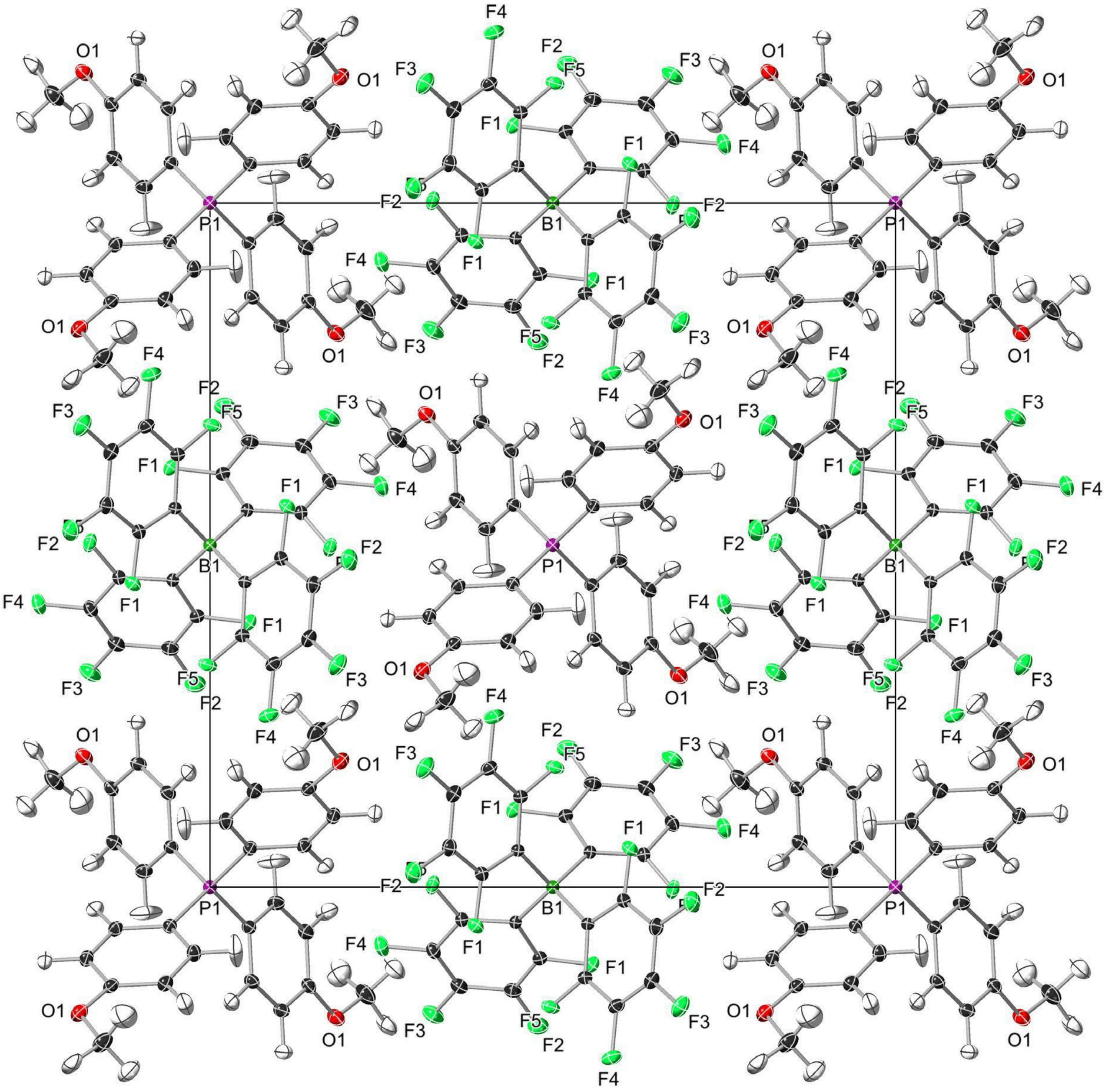

Figure S2: GM-B157 Packing

Conductivity Measurements were made with a YSI 3200 conductivity bridge utilizing a YSI 3256 dip probe with a cell constant of 0.091 (0.004 cm-1). The cell constant was calculated by repeat measurements of aqueous KCl solutions at two concentrations. The test solution was contained in a conductivity cell made by "sawing off" the top half of a 100-mL graduated cylinder. The diameter of this cell is normally set slightly larger than that of the YSI 3256 dip probe, thus minimizing the amount of solvent necessary for the experiment. Typically, 30 mL of solution were added to the conductivity cell and then stirred. The YSI 3256 contains a thermistor, and the temperature of the solution was recorded during each experiment. The temperature throughout an experiment remained consistent while the average temperature throughout the entire set of data was set to 22.45 (0.95 °C). Ten 100-μL aliquots of a nominally 3 mM stock solution of the electrolyte were added to the conductivity cell resulting in concentrations ranging from 10-5 to 10-6 M. After measurement of the final solution, the volume of solution in the conductivity cell was determined via the graduations on the graduated cylinder. In the case of tetrahydrofuran, the volume of stock solution added (1 mL) was compensated for by solvent evaporation.

A YSI 3200 Conductivity Meter with a model 3265 flow cell was used to take the conductivity measurements of TAP^R-TFAB (R= *p*OCH_3), TBA-TFAB and TBA-PF_6. Lee Wheaton curve fitting function was used to determine both K_a and $\Lambda 0$ values. Igor was used to analyze all of the data (**Table S2**).

Table S2. Conductivity data for TAP^R-TFAB salts in THF.

TAP-TFAB Substituents	Disassociation Constant (K_d)	Limiting Conductivity (Λ_0)	Association Constant
R=pOMe	3.03. X 10^{-4}	78.6 Scm^2/mole	3.30 x 10^3
TBA-TFAB	1.63 X 10^{-4}	86.2 Scm^2/mole	6.15 x 10^3
TBA-PF_6	2.86 X 10^{-6}	86.2 Scm^2/mole	37.13 x 10^3

These conductivity results show that the highest limiting conductivity of the synthesized salts belongs to TAP^R-TFAB (R=*p*OMe) at 78.6 S/m which is closest to TBA-PF_6, the leading industrial supporting electrolyte.

We performed electrochemical experiments to probe the effect of both the solvent and the synthesized electrolyte TAP^R-TFAB ion-pair (R= *p*OCH_3) as well as TBA-TFAB and TBA-PF_6 on conductivity in low-polar media (tetrahydrofuran THF and tert-butyl methyl ether TBME). Data for $TA^{OMe}P^+TFAB^-$ in THF correlate well with preliminary data: the values for Λ_0 and K_A were 90.1 $Scm^2\ mol^{-1}$ and 4.37 x 10^3. In TBME, the value for Λ_0 was lower than predicted by the Walden product (113 $Scm^2\ mol^{-1}$).

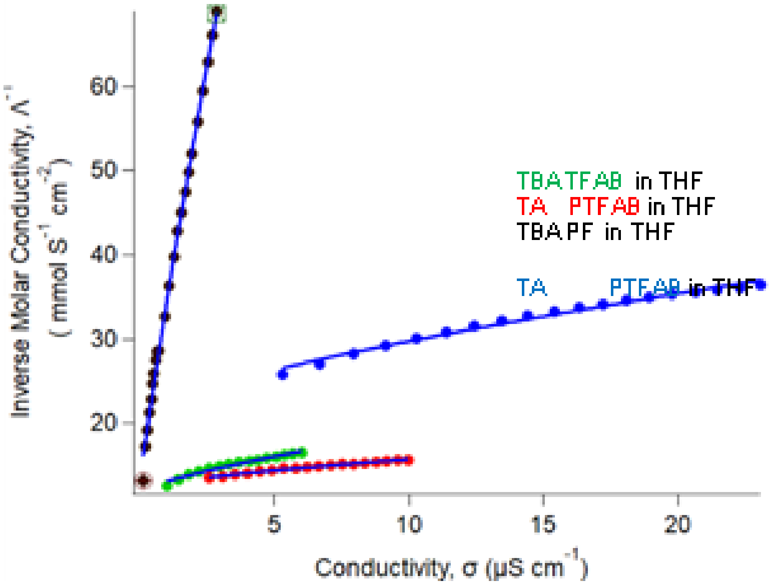

Figure S3: Inverse molar conductivity v conductivity of different electrolytes in THF

TBA^+TFAB^- *$TA^{OMe}PTFAB$* *$TBA^+PF_6^-$* and *$TA^{3,4\text{-}diOMe}PTFAB$*

There's a reduction in the values of association constant K_A from TBA^+TFAB^- electrolyte to the proposed electrolyte $TA^{OMe}P^+$ $TFAB^-$ in THF (**Figure S3**). This result is promising as it suggests that the novel $TA^{OMe}P^+TFAB^-$ salt is formed by a anion and a cation with a much weaker ion pairing ability than the established electrolyte TBA^+TFAB^-. A decrease in the K_A values from TBA^+TFAB^- to our synthetic $TA^{OMe}P^+$ $TFAB^-$ is also observed in the TBME conductivity tests shown below (**Figure S4**) further confirming $TA^{OMe}P^+TFAB^-$ as a more weakly coordinating electrolyte than TBA^+TFAB^-.

The strategy of increasing the size and bulkiness of the cation of the ion-pair seems to correlate well with a decrease in ion-pairing and an increase in limiting conductivity of the ion-pair in these low-polarity solvents.

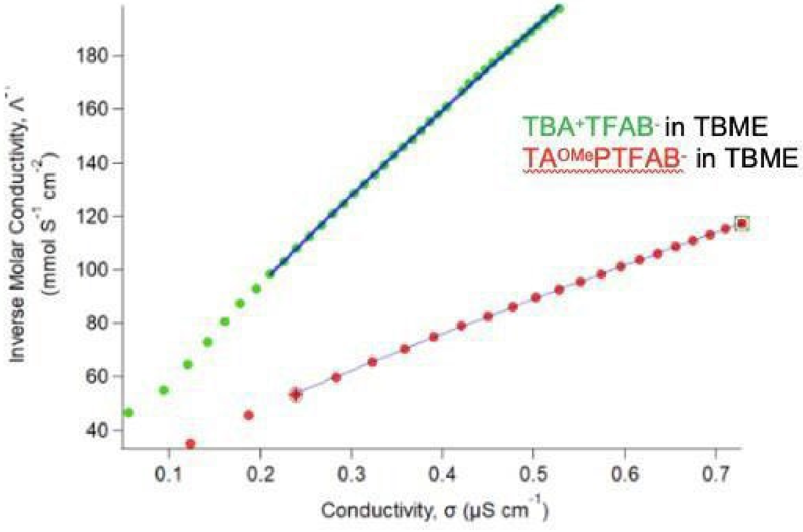

Figure S4: Inverse molar conductivity v conductivity of different electrolytes in TBME ***TBA/TFAB*** **and** ***$TA^{OMe}P/TFAB$***

NMR Spectroscopy and Mass Spectrometry Data

All NMR data were initially acquired at BNL, Upton NY and at UCONN, Storrs CT in 2018-2019. Soon after all restrictions related to the Covid-19 pandemic were lifted, new NMR data were recorded at USF-Tampa FL NMR facility using a 600 MHz Agilent spectrometer. High resolution mass spectrometry were acquired at the University of South Carolina (USC) Mass Spectrometry facility in Columbia SC. Single crystal X-ray crystallography data were acquired at Emory University, Atlanta GA.

Tetrakis(p-methoxy-phenyl) phosphonium bromide ***2 (P1)***

To an oven-dried 50-mL round-bottomed flask containing a magnetic stir bar, a reflux condenser fitted with a $CaCl_2$ drying tube, *p*-bromoanisole (1 mmol – 0.19g) was dissolved in *o*-xylene (20 mL), followed by the addition of tris(*p*-methoxyphenyl) phosphine (1.1 mmol – 0.39g) and of tris- palladium dibenziledene (5% mol – 0.050g). The reaction mixture was heated to reflux using a heating mantle for three hours, and then cooled briefly by removal from the heating mantle. Additional tris-palladium dibenziledene catalyst (5% mol – 0.050g) were added and the reaction mixture was refluxed for another 3 hr. The reaction mixture was cooled to room temperature and vacuum filtered using hexane to wash the residue (3x). The dry crude product which crystallized in the mother-liquor was further purified by recrystallization in hot ethanol yielding 0.52g (90%) of the phosphonium bromide **2**.

^{1}H NMR (600 MHz, $CDCl_3$) δ ppm: 3.77 (s, 3H, OCH_3), 6.89 (d, J = 7Hz, 2H, Ar-H),

7.49 (dd, J = 7Hz, 2H, Ar-H). **Figure S5**.

^{13}C NMR: (600 MHz, $CDCl_3$) δ ppm 55.35 (s, OCH_3), 113.96 (s, Ar-CH.), 123.96 (s, Ar-Cq.), 124.70 (s, Ar-Cq.), 133.94 (s, Ar-CH), 162.36 (s, Ar-Cq.). **Figure S6**.

^{31}P NMR (600 MHz, $CDCl_3$) δ ppm: 31.73 (s, (Br-**P**(Ar^{OMe})$_4$). **Figure S7**.

MS: TOF MS ES: m/z 459.17 $[M - Br]^+$. **Figure S8**.

Tetrakis(p-methoxy-phenyl) phosphonium- tetrakis(pentafluorophenyl)borate salt (TAP^R/TFAB, with R= p-OMe) ***3 (ZW1).***

To an oven dried 50-mL round-bottomed flask, containing a magnetic stir bar, a reflux condenser fitted with a $CaCl_2$ drying tube, *phosphonium bromide* ***2*** (1 mmol – 0.54g) was dissolved in *methanol* (20 mL), and lithium tetrakis(pentafluorophenyl)borate LiTFAB was added (1 mmol – 0.69g). The reaction mixture was gently heated to 70 °C using a heating mantle for 3 hr. The reaction mixture was cooled to room temperature and the precipitate was vacuum filtered using ice-cold methanol to wash the crystals (3x). The dry product was collected in the filter paper without further purification. Yield: ~1.0g (88%) of crude product, recrystallized in hot ethanol to yield of needle-like white crystals (0.25g) as first batch of high purity salt **3**.

^{1}H NMR (600 MHz, $CDCl_3$) δ ppm: 3.90 (s, 3H, OCH_3), 7.11 (d, J = 7Hz, 2H, Ar-H), 7.45 (d, J = 7Hz, 2H, Ar-H). **Figure S9**.

^{13}C NMR: (600 MHz, $CDCl_3$) δ ppm 55.79 (s, OCH_3), 108.63 (s, Ar-Cq.), 109.33 (s, Ar-Cq.), 116.08 (s, Ar-CH), 135.90 (s, Ar-CH), 147.32 (s, Ar-Cq., perfluorinated ring), 148.94 (s, Ar-Cq., perfluorinated ring); 164.99 (s, Ar-Cq. phosphonium). **Figure S10**.

^{19}F NMR: (400 MHz, $CDCl_3$) -132.7 (s, 2 Ar-F, *m*), -163.1 (s, 1 Ar-F, *p*), -167.0 (s, 2 Ar-F, *o*). **Figure S11**.

^{31}P NMR (600 MHz, $CDCl_3$) δ ppm: 20.68 (s, $(^FAr')_4$ B--**P**$(Ar^{OMe})_4$). **Figure S12.**

MS: Positive ESI EIC: m/z 459.17 $[M + H]^+$ for the cation TAP^{OMe}. **Figure S13.**

Negative ESI EIC: m/z 678.98 $[M + H]^-$ for the anion TFAB. **Figure S14.**

NMR and HRMS DATA

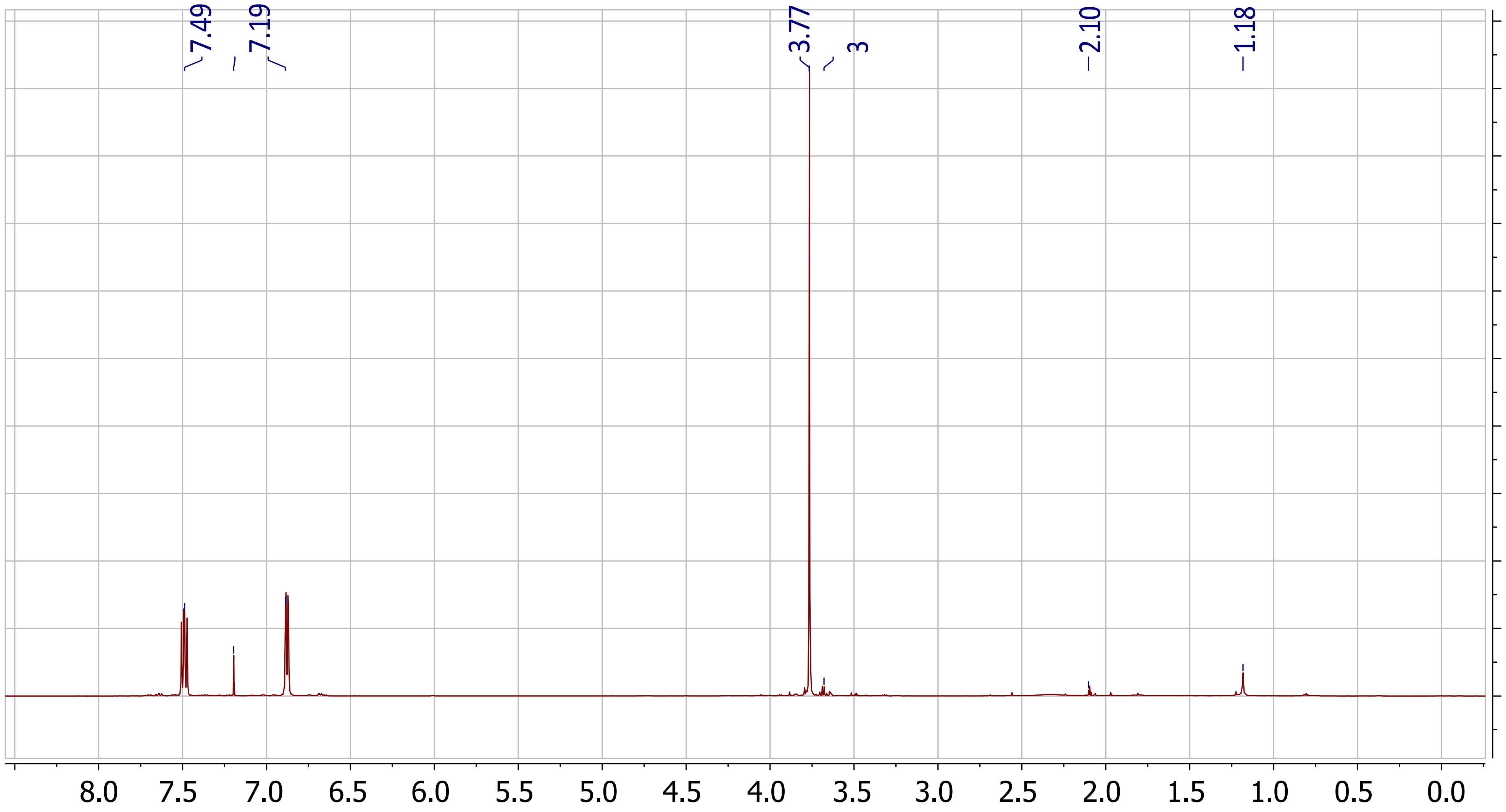

Figure S5. ^{1}H NMR Spectrum of **P1** in $CDCl_3$

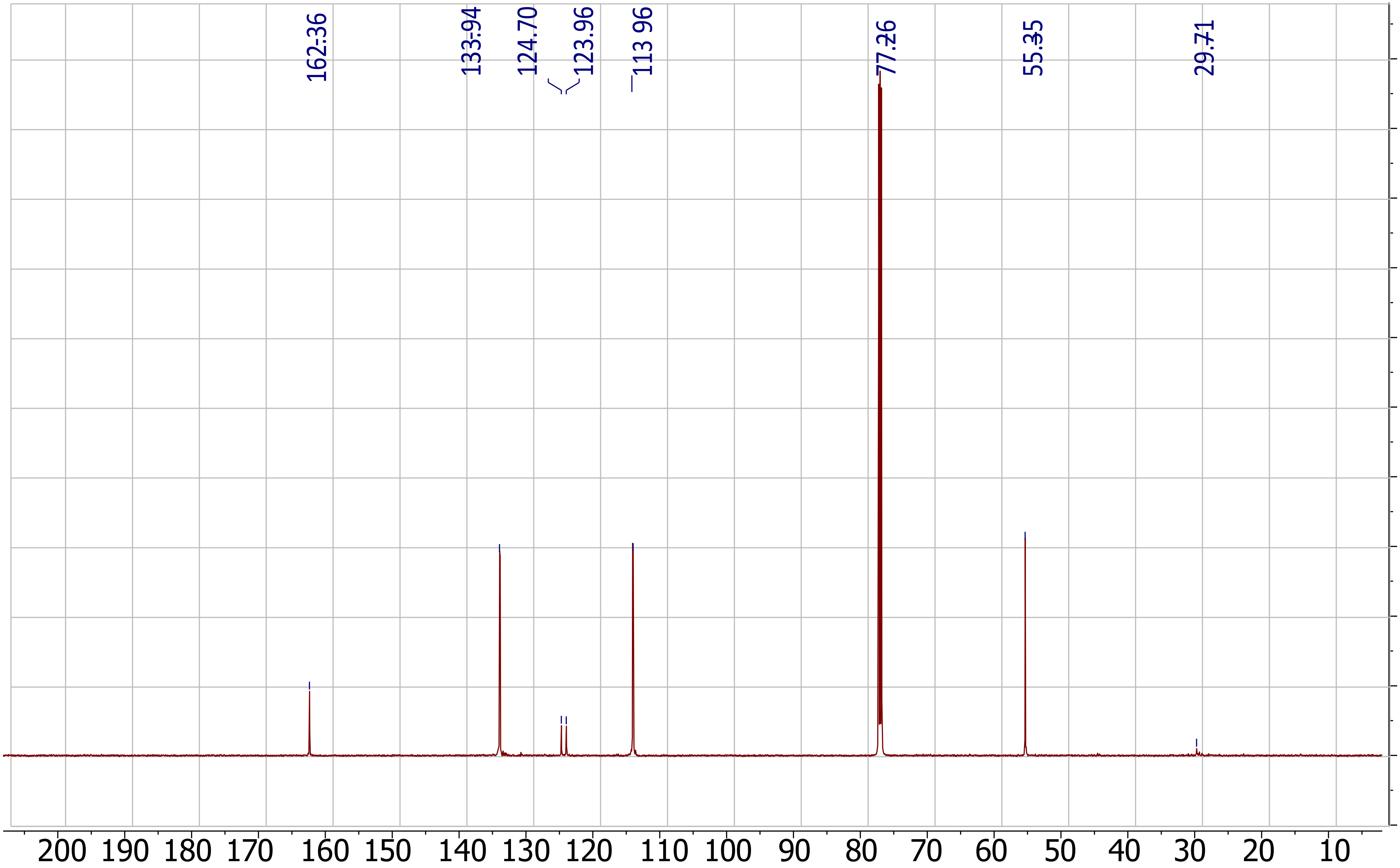

Figure S6. ^{13}C NMR spectrum of **P1** in CDC_3

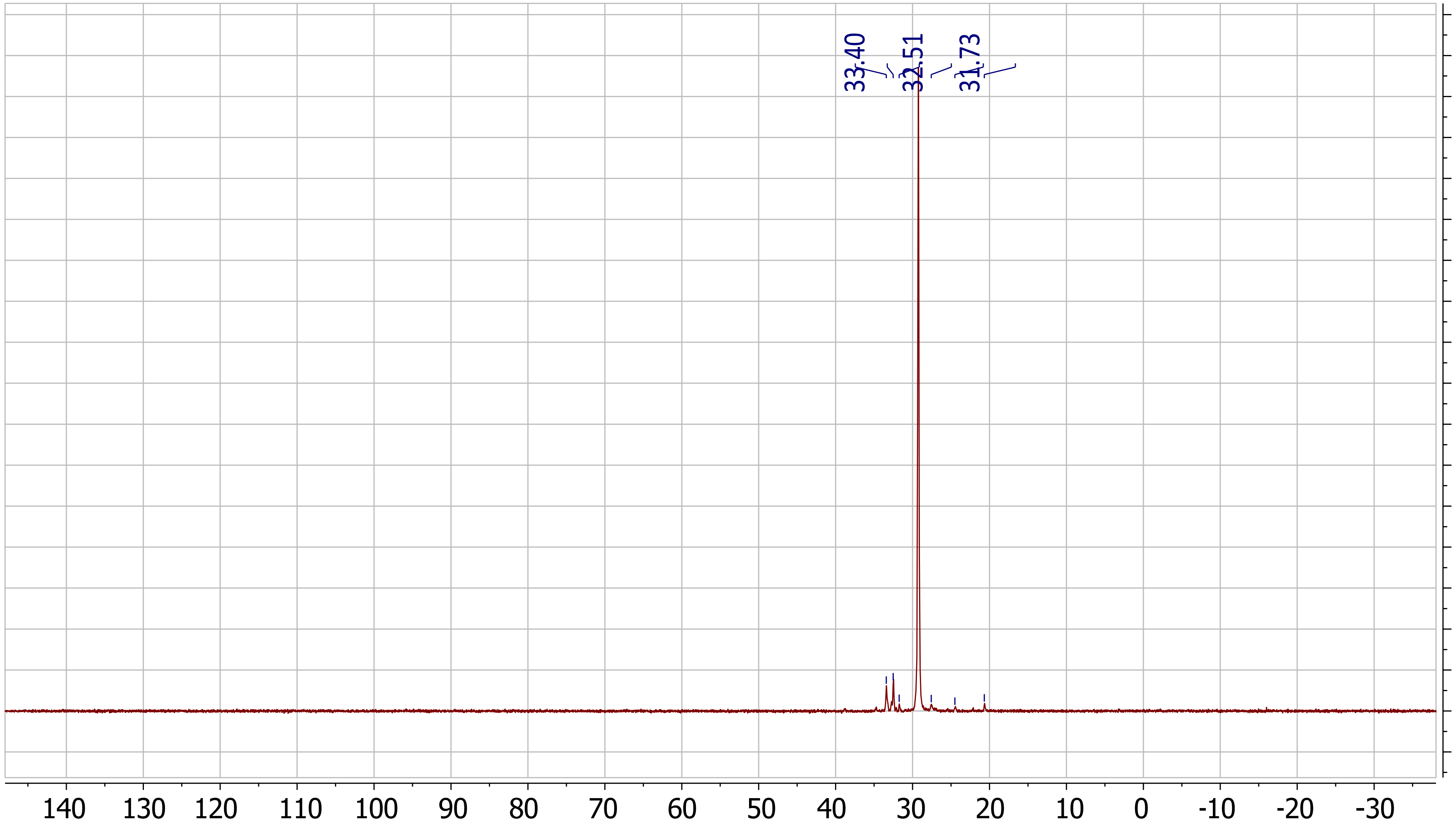

Figure S7: ^{31}P NMR Spectrum of **P1** in $CDCl_3$

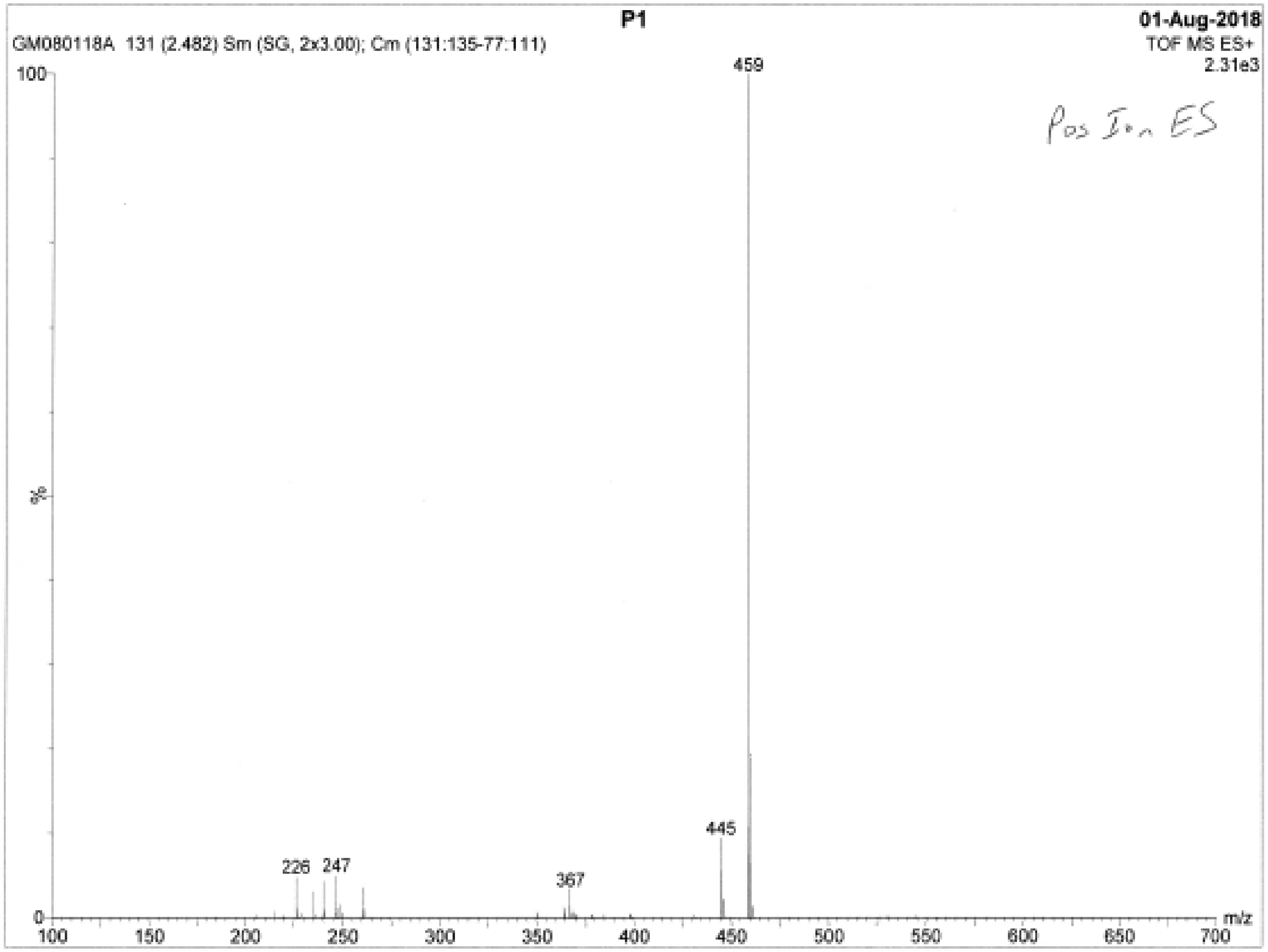

Figure S8: High Resolution Mass Spectrum of **P1**

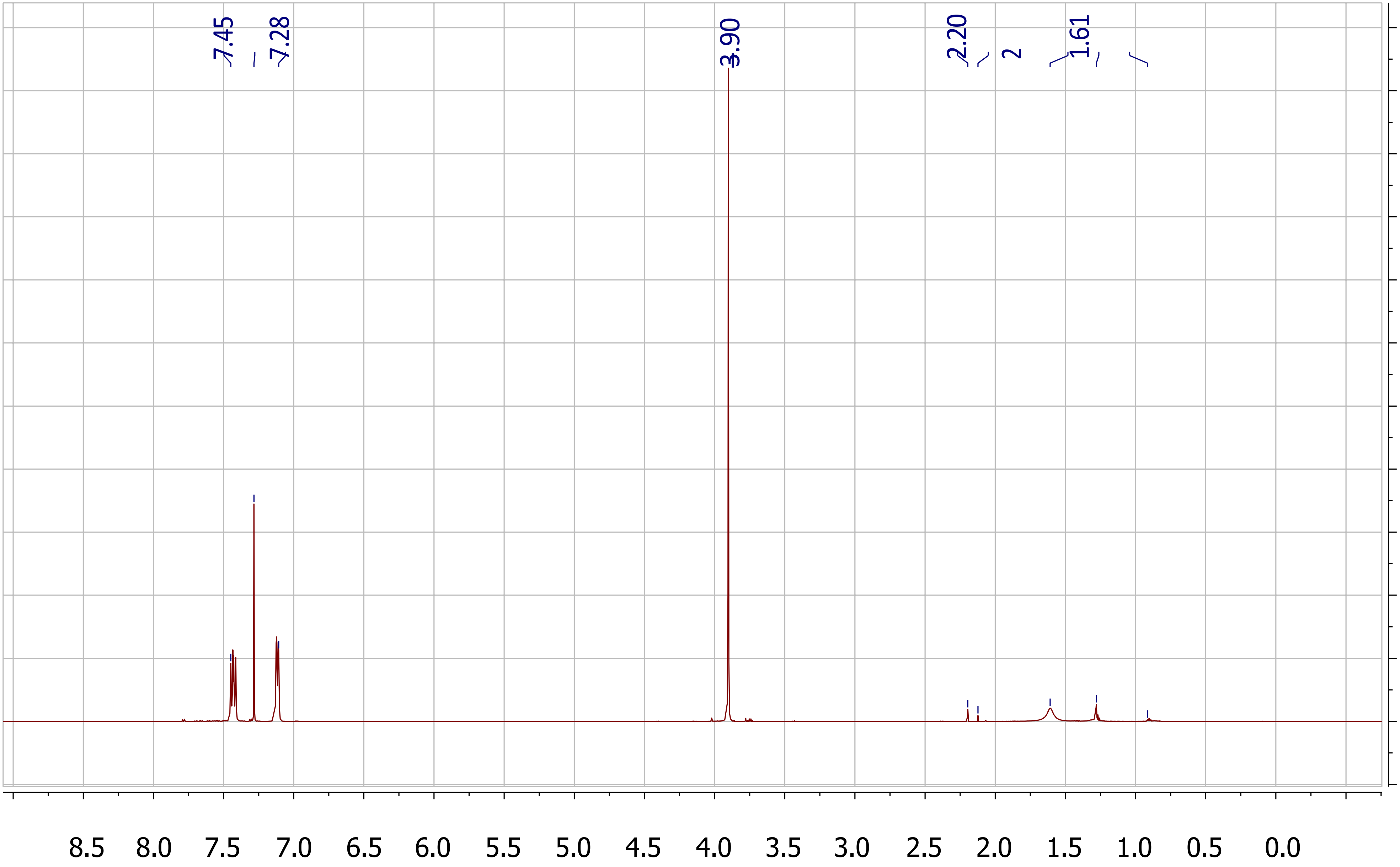

Figure S9: ^{1}H NMR Spectrum of **ZW1** in $CDCl_3$

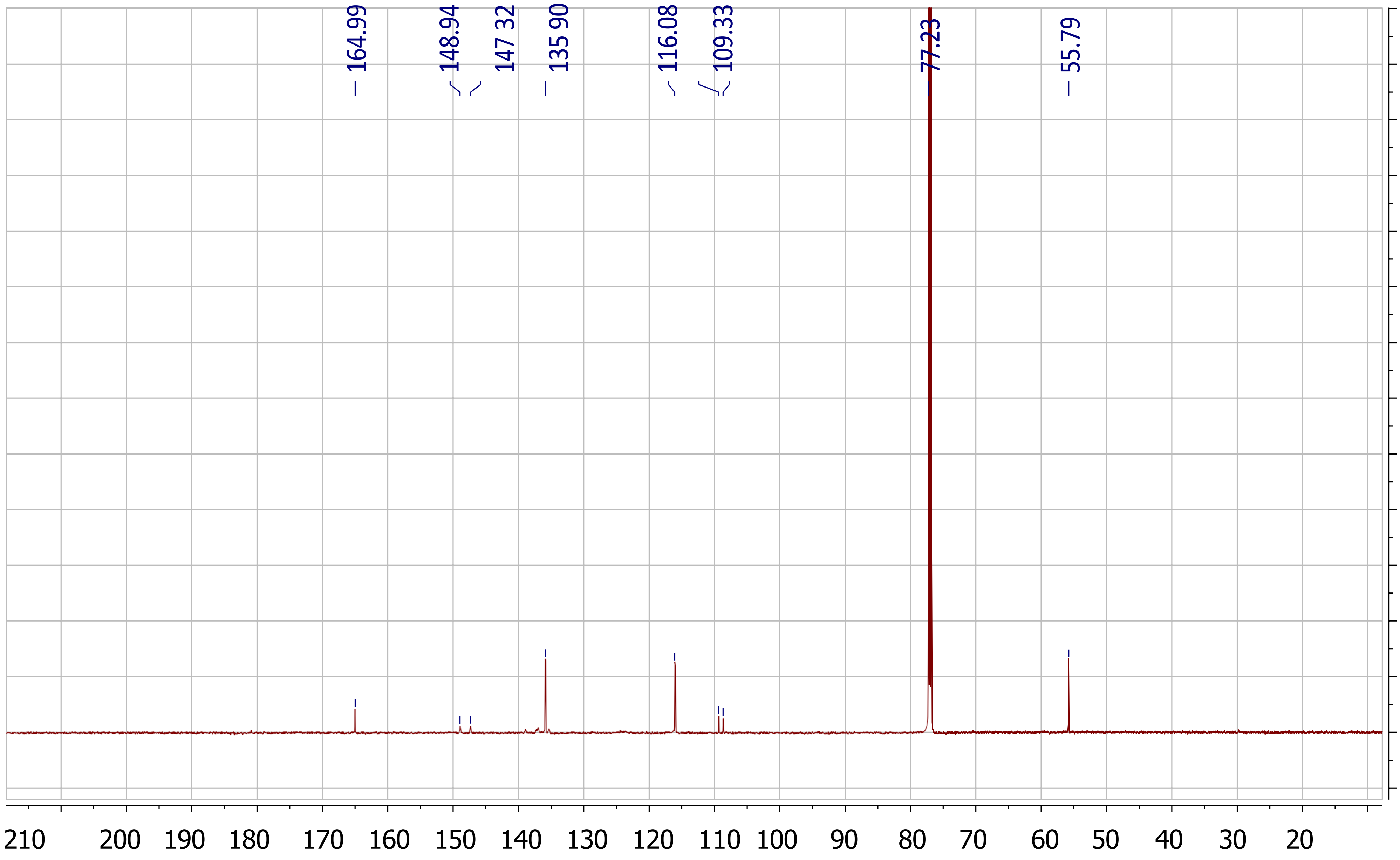

Figure S10: ^{13}C NMR of ZW1 in $CDCl_3$

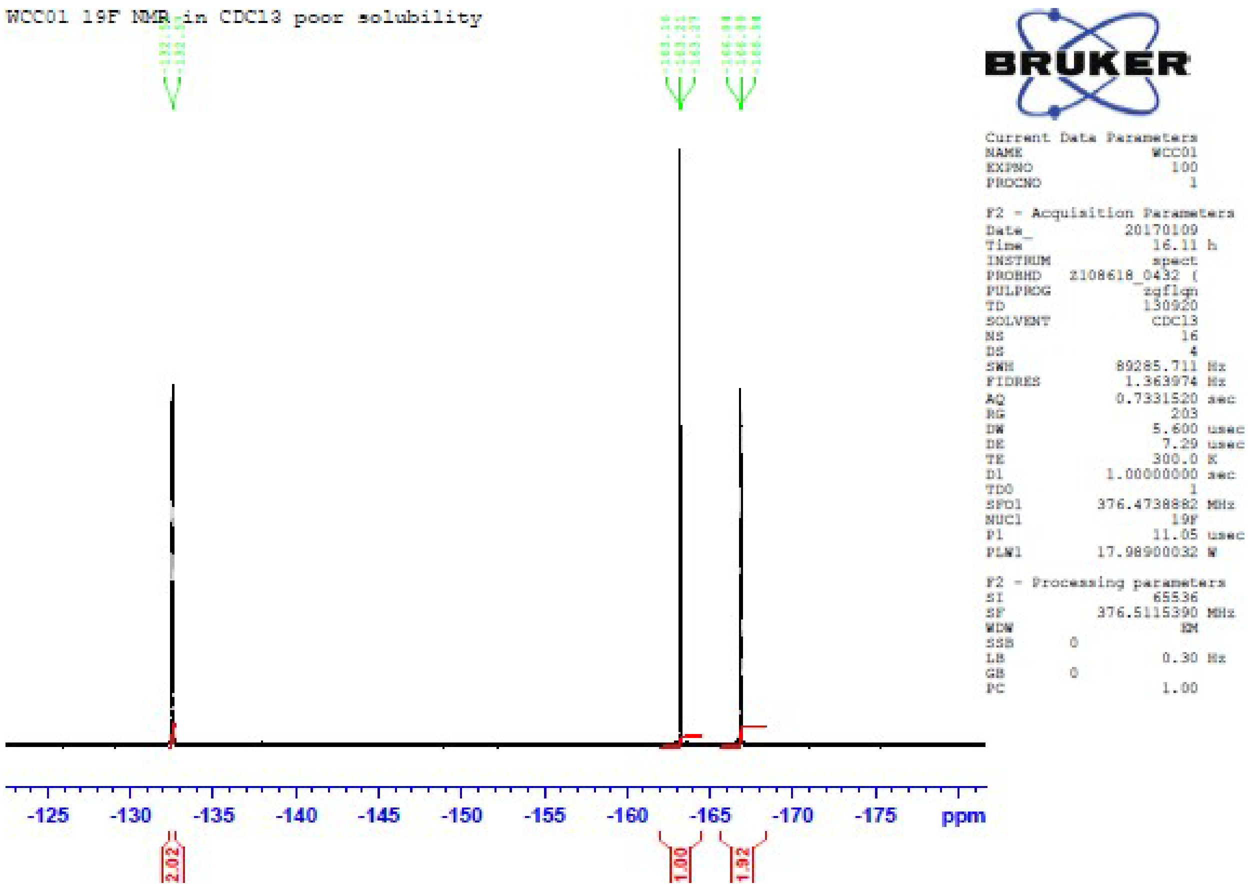

Figure S11: ^{19}F NMR Spectrum of ZW1 in $CDCl_3$

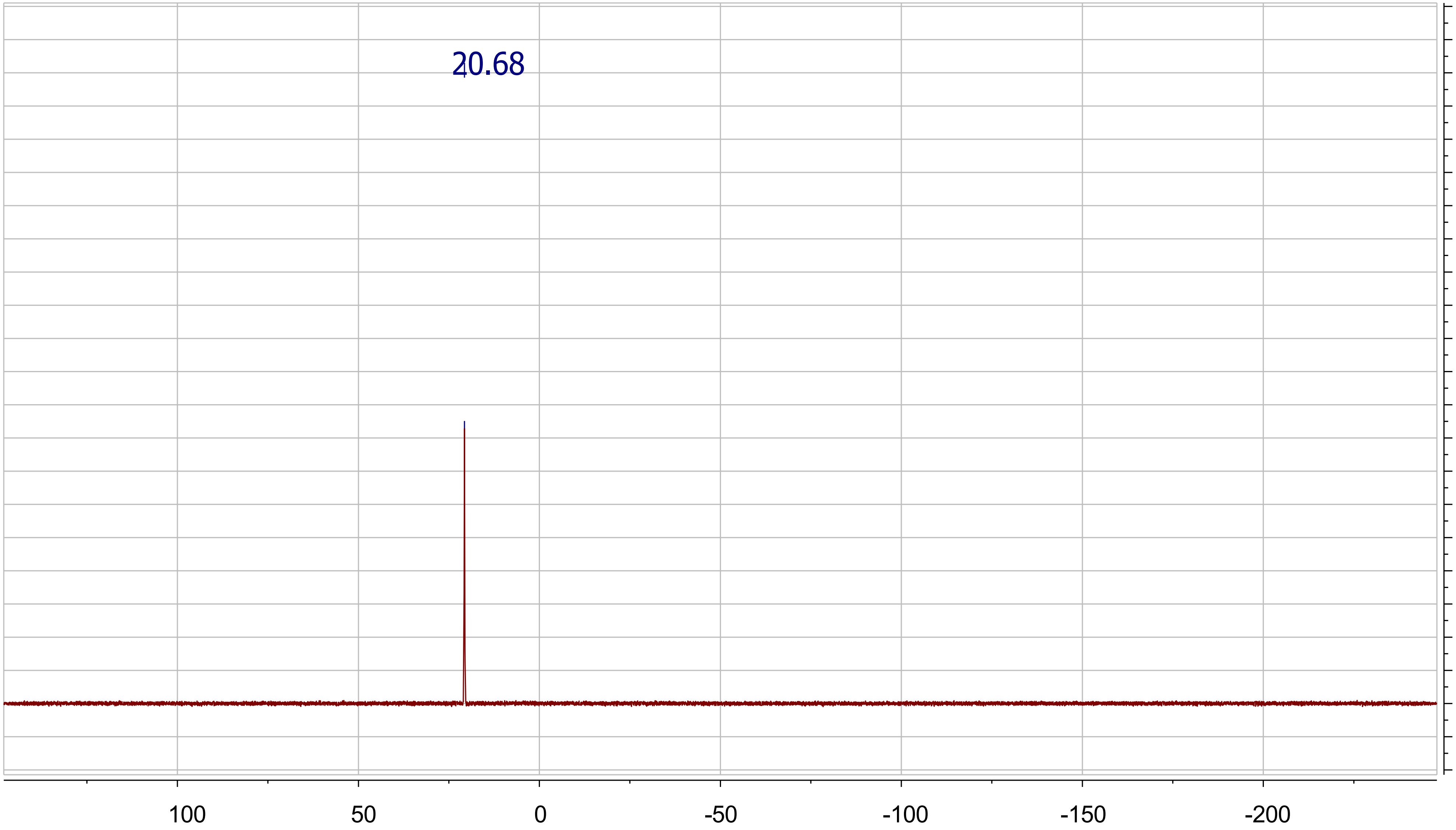

Figure S12: ^{31}P NMR Spectrum of ZW1 in $CDCl_3$

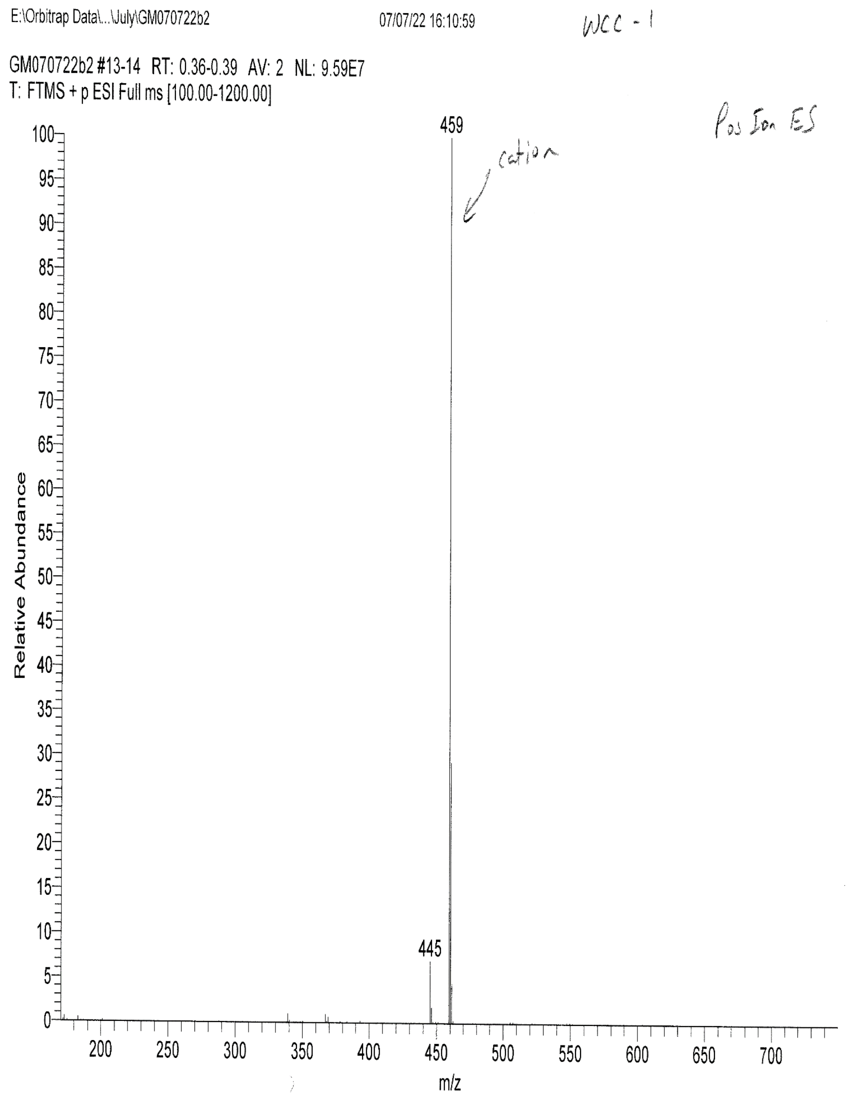

Figure S13. Mass spectra of the cation for ZW1

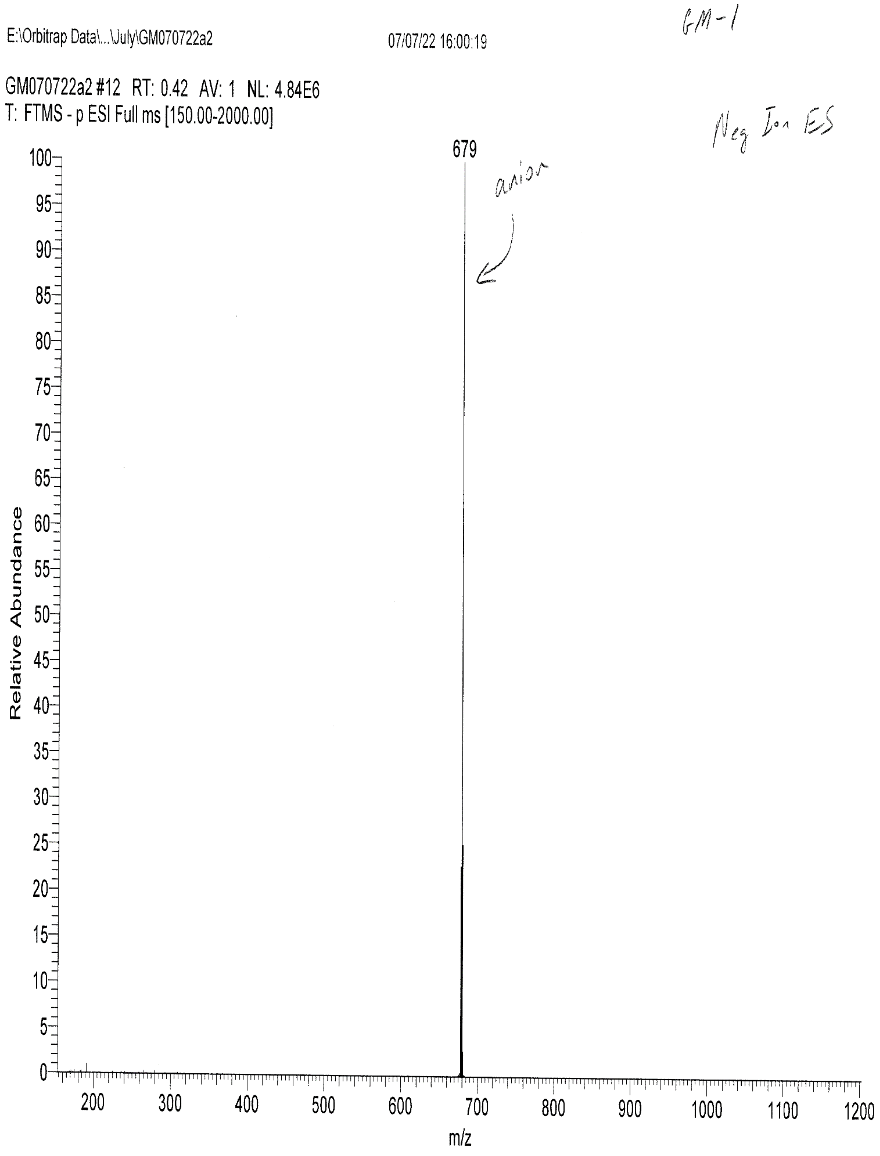

Figure S14. Mass spectra of the anion for ZW1

References

[1] Bruker, *SAINT*, *SADABS*, Bruker AXS Inc., Madison, Wisconsin, USA.

[2] Krause, L.; Herbst-Irmer, R.; Sheldrick, G.M.; Stalke, D. Comparison of silver and molybdenum microfocus X-ray sources for single-crystal structure determination, *J. Appl. Cryst.* **2015**, *48*, 3–10, doi:10.1107/S1600576714022985.

[3] Sheldrick, G.M.; A Short History of SHELX, *Acta Cryst.* **2008**, *A64*, 112–122, doi:10.1107/S0108767307043930.

[4] Sheldrick, G.M.; Crystal structure refinement with SHELXL; *Acta Cryst.* **2015**, *C71*, 3–8, doi:10.1107/S2053229614024218.

[5] Groom, C.R.; Bruno, I.J.; Lightfoot, M.P.; Ward, S.C.; The Cambridge Structural Database; *Acta Cryst.* **2016**, *B72*, 171–179, doi:10.1107/S2052520616003954.

[6] Kratzert, D.; *FinalCif*, *V107*, https://dkratzert.de/finalcif.html (accessed 2022-08-04)
